# American Dental Association

**Published:** 1890-10

**Authors:** 


### Reports of Society Meetings.

### AMERICAN DENTAL ASSOCIATION.—THIRTIETH ANNUAL MEETING, EXCELSIOR SPRINGS, MO., AUGUST 4 TO 8, 1890.

(Continued from page 538.)

*Tuesday Evening's Session.*

At the Tuesday evening session of the American Dental Association President Foster appointed L. D. Shepard, Boston, Mass.; W. W. Walker, New York; O. S. Hunt, Iowa City, Iowa; H. P. Noble, Washington, D. C.; and George W. McElhany, of Columbus, Georgia, a committee of conference upon the World's Fair matter. The Committee of Conference from the Southern Dental Association consisted of L. D. Carpenter, Atlanta, Ga.; J. Y. Crawford, Nashville, Tenn.; W. J. Barton, of Dallas, Texas; J. Taft, of Cincinnati, O.; and C. S. Stockton, of Newark, N. J.

The report of treasurer A. H. Fuller, of St. Louis, showed a balance on hand last year of $2028.53. The total receipts for the year amounted to $1080, and the total expenditures to $1739.81, leaving a balance on hand at the present time of $1368.74.

Section 5 was first called, but owing to the absence of the chairman, Dr. A. W. Harlan, it was passed. All of the other sections were then passed until Section 2 was reached. Dr. C. N. Peirce, chairman, presented the paper of Dr. W. H. Atkinson, of New York, for reading and discussion. The title of the paper was "Education, Nomenclature, and Terminology." After stating that anything like an exhaustive demonstrative presentment of the subject in the brief period allotted to essayists was impossible, Dr. Atkinson said,—

The term education signifies to draw out of the storehouse of the mind demonstrations capable of being formulated and held in consciousness ready for use in the efforts to communicate in a satisfactory manner the mental processes through which they have been acquired. This involves a comprehension of the work in progress to the completion of the stages of mental growth through which we are carried to attain a full understanding of the processes pertaining to the healing of mind and body.

The naming of these stages would properly be the work of nomenclature or the classification of things.

The technique of recording various movements involves the proper arrangements of terms which are the elements in terminology upon which correct classification depends.

The greatest obstacle in the way of the teacher is the assumption of finality, requiring the learner to refer and defer to the recorded statements in the earlier states of the records. By this saying I do not mean to ignore the potency of memorized trends of thought, but I do mean to advocate the necessity of keeping in touch with the present illumination of learners and teachers throughout the whole range of valuable and minute research.

The particular instance that comes to my mind now as specially obstructive to our progress is the attributing of the power which operates the function to the body at which the changes occur. For instance, it is said of the liver that it secretes bile, which is not true. Bile is secreted in the liver by a process not yet demonstratively formulated. Probably the greatest mistake is made in saying, Give me the measure of phosphorus on a planet and I will give you the amount of mental activity possible to that planet; which is often formulated in a modification of this statement by saying, The brain secretes thought, and where no brain is there is lack of mental action, and such like statements. All such naturalists fail to see that the power is before and in all of its manifestations, the justification of which has already been stated in this paper,—viz., the physiology of the mineral kingdom is crystallization, that of the vegetable kingdom is cellulation, that of the animal kingdom is corpusculation.

A friend asks, What is the significance of the skin ? and probably had little conception of the reach of such a query. Take a piece of human skin and study it minutely, and it will be found to consist of an outer surface and an attached surface to the body of the organ of the body constituting the system.

The principal function of the skin and the mucous membrane is depurative,—that is to say, the throwing out of the excess of pabulum, effete matter, and *débris* resultant upon functional activity of the underlying organs. The mucous membrane normally absorbs nutrient material when reduced to the character of a perfect peptone, and under certain circumstances the skin may in a minor degree do the same when immersed in a solution where perfect peptones abound.

All this and more has to be stored in the mind to avail ourselves of the best modes of education. The stored mental activities of ancestry is one measure of individual endowment. This hereditary endowment of past molecular experiences and effectional activities of ancestry lays the foundation, by involution, of the possibilities of evolution, of which so much is made in these days of scientific inquiry as to the origin and development of species and varieties. I have seen children for whose mothers I have performed the functions of an obstetrician which I recognized as belonging to the family by their manner of locomotion when I only saw their backs; and if in one man's experience of a short career there shall be a multiplicity of examples pointing to this doctrine, it will not be deemed hallucination or dementia to state that the agreements and disagreements and the quarrels indulged in by the parents are visited upon their children, as the Scriptures say, to the third and fourth generation. Then let those teachers who love their calling and their pupils take courage and go forward.

Had we the record of all the past condensed into the single syllabus of one paragraph, we would be minus of the accomplishment of our desire to lead our progeny into a fuller appreciation and understanding of the problems of life.

In closing, Dr. Atkinson referred to the methods of work and the presentation of papers to the American Dental Association. If progress and improvement are the objects aimed at, he insisted that the members of the society should be wide awake to the opportunities presented. He urged a greater esteem for the life-work committed to the charge of all. He advised all to go forward and meet the future bravely and to attempt the getting at the bottom of things. He advised that all papers presented to the society should be fresh, thoughtful, and vigorous.

Dr. A. H. Thompson, of Topeka, Kan., read a paper on "Scientific Education in our Colleges." He said,—

That scientific education is in danger there is no doubt. The practical man insists that students should hurry on to money-making, the chief aim and end of the life of the modern. His gospel is purely commercial, his instincts are purely financial, and he believes that science is fit only for cranks or for the curious. The plea of this paper is for a scientific education pure and simple. The ground is taken that the student should love learning for its ennobling and elevating powers, and not because it brings increased facilities for making money. A Nemesis of money-making broods over the land like a death damp. This spirit has prostituted science until nothing is thought of except the acquisition of the almighty dollar. In this there is nothing new. The money-makers are as old as the race. But it is the apparent triumph that alarms us. The practical man has always pleaded for more artisans, more industrial schools, an increased material education. But in spite of the practical man science is advancing and will continue to advance. The creed of money-making is in the very air that we breathe, and there is need for some antidote. But few men have escaped from the fever of wealth-gathering. Few men are wise in this respect, and but few have learned to rate wealth at its true value. The true student has learned how to get out the best there is in life. He is in touch with the ages, and is loyal to truth and to science.

We would not be ungrateful to the artisan. We glory in our material advancement. There are noble men who wear the garb of mechanics. Artistic operating is well and good in its way, but it is not necessary that applied science should always be placed upon such a lofty pedestal. The basis of action is knowledge. If we are to train our boys and girls only in the arts the apprenticeship system would be the better and the easier. Why should the practical man wish to degrade science? It has been his best friend and has kept alive the spirit of research in the world. The office system is the delight of the practical man. There he reduces things to a system subject to the one will of the master. After all, the foundations of science never change. Practice is science materialized, therefore science is a practical thing in itself. Yet with the materialization of science has come the materialization of civilization. By thought man seeks out secret forces and powers. By thought he defies disease and conquers nature and the natural. But the savage is pre-eminently practical.

In dentistry we are entering upon a new era. The three years' course in our colleges is about to be made general. The main cause of the lowering of the standard heretofore has been found in the fact that it was necessary to bring down the subjects to so low a platform that they could be mastered by the average dental student. Now we have more time for the work of preparation. Let us lay the foundations deep and broad during the first year. The second year the lectures should be largely didactic. The third year should be more comprehensive in its scope, but throughout the entire course we should keep in mind the principles of simplicity and precision. It would be a great mistake to put the fundamentals out of sight. What we want is a series of monographs so arranged that the students will be able to master the main points.

The great weakness of our teachers has been that they have not been trained in the art of teaching. For a busy practitioner to rush from his work to the school-room and deliver a lecture without preparation is to invite careless work on the part of the students. We need teachers born to the work, teachers who will take the time to study and to prepare their lectures.

The manual training movement, which is attracting so much attention in this country, should be confined to the primary courses. If the intention and the object is to make athletes and artisans of our boys, it will be better to have none of it.

Referring to the conflict between the State Boards and the dental colleges Dr. Thompson said,—

The State Boards must be made up of men who are skilful and well educated, both in theory and practice. If not prepared in the details of the various branches they should employ specialists. The colleges will follow where the boards lead. The boards are in authority, they are custodians, and with them rests in a large measure the standard of the future. We believe in the ultimate triumph of the right. We have confidence in the strong man. The weak man is the superficial man. A profession is scientific, a trade materialistic. Let us guard the sciences closely, well remembering that if we lose sight of knowledge we lose sight of all that is valuable.

A paper written by Dr. Charles B. Atkinson on "Education and the Obligations it involves" was next presented by Chairman C. N. Peirce. The writer commenced with a discussion of the duties and obligations of the educated. He held that the truly educated man will not spend too much of his time up in the clouds. This is not a danger that besets the practical mind. The cry of modern dentists is for greater and greater skill. The true training of the student should be a combination of the theoretical and the practical. If theory is indulged in to a great extent the very theories give rise to many difficulties. Each chair in the various schools thinks it necessary to propagate a new theory. Each school has its specialties, and those in authority place special stress upon certain teachings emanating from the various faculties. But a broader and better course would indicate a training more general. It is not necessary to find fault with our colleges all the time, but there should be a change in methods. It would be considerable trouble to rewrite lectures every year, but why should not the students have the advantages of the improvements going on in the dental world? Why is it that wholesale extraction is still practised to such a large extent among the dentists of to-day? Who is to blame? When George Quackenbos Coulton told the story of three thousand teeth extracted, before a large and brilliant audience of dentists, only one voice was raised against the practice. Men who work by the hour are mechanics. They should go into the shops. There are too many dentists to-day who are conducting their offices upon the factory basis.

Then how about ethics? Have we a code of ethics as a profession? The man who owes a debt to one dentist never has any trouble in contracting debts with another dentist. We talk of our profession. There is such a thing as being a trader. The colleges claim that they do the best that is possible with the material presented. One plan would be to have the applicants to our colleges examined as to moral, mental, and mechanical fitness. There is little need in life at the present time for the men who can only extract, but cannot save teeth. Then, again, too many men go into literature and into the societies for advertising purposes. Some men seek preferment, and too often they find it. The few bear the brunt of the work, the others gather in the glory. After all, the United States has many names that should live forever. They have honored their country and their profession. Some are dead, some are still living. They will be remembered, but how small the satisfaction of saying, "I will be remembered when dead." The field of ethics is a broad one. True worth should be the criterion. In our societies wise counsels should prevail. The occupancy of an office should carry with it the dignity and responsibility of office. The names most illustrious in the history of the profession have been the names of men who have carried on the warfare with disease, not merely for a livelihood, but because of their love for the profession. The three years of six months each now required of our graduates should give us better men. A thorough education will tend to equalize the character of the graduates and pave the way for uniform laws. Then a code of ethics, based upon common sense and common honesty, will not be ignored.

A paper written by Dr. B. A. R. Ottolengui, of New York, was next presented. The subject of the paper was a "National Dental Education." The writer based his objections to the present system of dental colleges upon the following points:

1. Each school has a standard of its own.

2. The professors and teachers are interested commercially in the success of the schools.

3. Junior and senior classes are obliged to listen to the same lectures.

4. The classes are so large that only the persistent student learns.

The writer advocated a national dental degree, and insisted that this would serve as a basis of comparison to determine the merits of the different schools. He held that a national dental university could not be expected this century. For the present a national board of dentistry could be created with power to grant the degree of D.O.S., or Doctor of Oral Surgery. He would have this board meet alternately on the Atlantic coast, in the valley of the Mississippi, and on the Pacific slope. The theoretical examinations could easily be conducted by the board, the technical examinations could be conducted through clinics. An examination fee should be charged to go to the treasury of the board. This fee should be variable, and should discriminate in favor of the students coming from a distance. It would be necessary to keep before the public, through the secular papers, the fact that examinations of this nature are being conducted, as well as the fact that a higher degree is conferred upon worthy students in the fields of dentistry, in honor of their work. The writer held that the point so long aimed at would soon be reached, and the various colleges would boast not how many students have been graduated from the respective institutions, but of the number that have attained to this higher degree. In explanation of the good that a board of this nature would do, the writer cited the example of the State Examining Board of New York. Since 1870 two hundred and ten men have applied for certificates. Only one hundred and three have succeeded. Of the graduates from the dental schools, twenty-two out of twenty-five have passed, and consequently certificates from these colleges have been at a premium. The writer was emphatic in his demands for legal enactments, as well as for a strong moral force, to back up the laws of the States.

The question was asked by one of the members of the Association, if a paper very much like this had not been presented in the Chicago *Dental Review.* The title of the paper was given as "Looking Forward." The chairman explained that the writer of the paper had given credit for the passages taken from the paper referred to, and that in accordance with the rule of the Association requiring original matter the quotations had been omitted in the reading.

The convention than adjourned until Wednesday morning.

(To be continued.)

